# Maker Buoy Variants for Water Level Monitoring and Tracking Drifting Objects in Remote Areas of Greenland

**DOI:** 10.3390/s20051254

**Published:** 2020-02-25

**Authors:** Daniel F. Carlson, Wayne J. Pavalko, Dorthe Petersen, Martin Olsen, Andreas E. Hass

**Affiliations:** 1 Arctic Research Centre, Department of Bioscience, Aarhus University, 8000 Aarhus, Denmark; 2 Institute of Coastal Research, Helmholtz-Zentrum Geesthacht, Centre for Materials and Coastal Research, 21502 Geesthacht, Germany; 3 WRG LLC, 12169 Flowing Water Trail, Clarksville, MD 21029 USA; wayne.pavalko@jhuapl.edu; 4 Asiaq Greenland Survey, Qatserisut 8, 3900 Nuuk, Greenland; dop@asiaq.gl (D.P.); mno@asiaq.gl (M.O.); AEH@asiaq.gl (A.E.H.)

**Keywords:** Greenland, glacial lake outburst flooding, iceberg tracking, environmental monitoring, affordable sensors, real-time data

## Abstract

Meltwater runoff from the Greenland Ice Sheet changes water levels in glacial lakes and can lead to glacial lake outburst flooding (GLOF) events that threaten lives and property. Icebergs produced at Greenland’s marine terminating glaciers drift into Baffin Bay and the North Atlantic, where they can threaten shipping and offshore installations. Thus, monitoring glacial lake water levels and the drift of icebergs can enhance safety and aid in the scientific studies of glacial hydrology and iceberg-ocean interactions. The Maker Buoy was originally designed as a low-cost and open source sensor to monitor surface ocean currents. The open source framework, low-cost components, rugged construction and affordable satellite data transmission capabilities make it easy to customize for environmental monitoring in remote areas and under harsh conditions. Here, we present two such Maker Buoy variants that were developed to monitor water level in an ice-infested glacial lake in southern Greenland and to track drifting icebergs and moorings in the Vaigat Strait (Northwest Greenland). We describe the construction of each design variant, methods to access data in the field without an internet connection, and deployments in Greenland in summer 2019. The successful deployments of each Maker Buoy variant suggest that they may also be useful in operational iceberg management strategies and in GLOF monitoring programs.

## 1. Introduction

Melting of the Greenland Ice Sheet (GrIS) has accelerated in recent years [1,2,3,4]. Meltwater can take multiple pathways to the ocean where it contributes to global sea level rise [5] and impacts stratification in fjord and shelf waters [6,7]. Ice-dammed lakes, fed by meltwater runoff, recurrently drain catastrophically through temporary subglacial conduits, resulting in glacial lake outburst flooding (GLOF) [1,8,9,10,11,12,13,14]. GLOF events can threaten lives and property [11,15,16] and also lead to rapid changes in temperature and salinity in proglacial fjord waters [17,18]. Glacial lakes are usually located in remote areas that are difficult to access and that lack basic infrastructure, like power and communications [10,19]. Glacial monitoring, therefore, relies heavily on satellite remote sensing data [12,20]. Past glacial lake monitoring programs have collected in situ data [21] and time lapse imagery [16], though few real-time early warning systems (EWS) have been reported [10,11]. A GLOF EWS in Greenland that is informed by real-time water level data would enhance public safety and safeguard critical infrastructure.

Icebergs account for approximately half of the freshwater flux from the GrIS to the ocean [22,23] and can impact ocean stratification [17] and marine ecosystems [24] while also threatening maritime shipping and offshore installations [25,26]. Despite the large number of icebergs in Greenland, little is known about their drift trajectories [27,28], deterioration rates [29], and the chemical and biological composition of the ice [24], due primarily to a lack of in situ observations. Much like glacial lakes, many of Greenland’s fjord systems are difficult and costly to access and are subject to highly variable weather and ice conditions. Furthermore, icebergs are unstable and may change orientation and/or capsize without warning [28,30], thereby presenting additional risks to scientific personnel and instrumentation. Similarly, oceanographic studies that deploy drifting instrumentation in remote fjords in Greenland require access to near-real-time position information to facilitate retrieval.

Clearly, scientific studies of GLOF and iceberg-ocean interactions could benefit from additional in situ observations, though appropriate sensors are either not commercially available or are not affordable on a meager research budget. Additionally, many commercially available global positioning system (GPS) tracking devices cannot be easily modified to include additional sensors [27], requiring specific components to be sourced for each use case. Many GPS trackers require an internet connection to view and download the data [27]. Researchers in remote areas of Greenland often lack mobile internet access, as most of Greenland is not permanently inhabited and cellular networks are limited to population centers. Similarly, satellite broadband internet is currently expensive, making low-cost, two-way satellite communications essential, as well as a field operable satellite data-link.

Environmental monitoring in Greenland could be simplified by using sensors that share common and open source hardware and software for general requirements like power, GPS, data storage, and communications. This device could then be customized to include appropriate sensors for each application, as needed. The total cost of such a sensor should also reflect the risks posed by ice and harsh environmental conditions. Given the costs of logistics in remote locations in Greenland, the size, weight, and power (SWAP) consumption should also be minimized. Researchers should be able to change the sampling rate to enable high frequency sampling during the summer melt season and lower frequency observations during winter.

We present two variants of the Maker Buoy (https://www.makerbuoy.com/) that were adapted to monitor water level in glacial lakes and to track drifting objects in Greenland. The Maker Buoy is a low cost ($500–$800, depending on optional components) and open source sensor that aligns well with the aforementioned measurement strategy. The Maker Buoy was originally developed to observe surface ocean currents and can be customized to integrate additional sensors. The Maker Buoy uses a low-cost iridium modem to enable two-way satellite communications. Data can be accessed in the field using a laptop computer and the same Iridium modem used in the Maker Buoy. This manuscript is organized as follows: In Section 2 we describe both variants of the Maker Buoy, data access and data management, and both study areas. Results are presented and discussed in Section 3.

## 2. Materials and Methods

### 2.1. Maker Buoy Design

**Figure 1:**
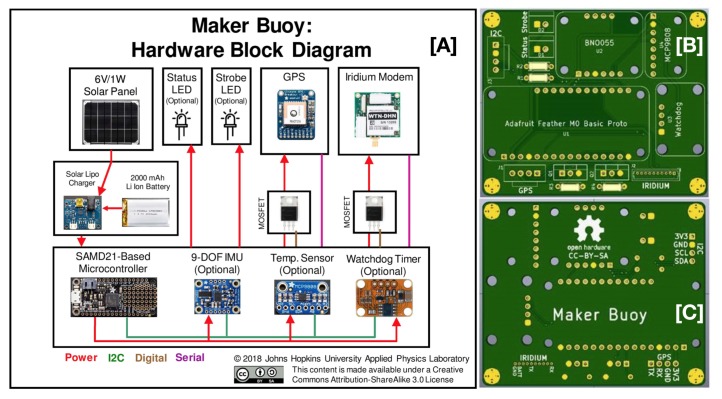
(**A**) A block diagram that shows the primary electronic components used in the Maker Buoy. (**B**) The front of the Maker Buoy PCB. (**C**) The back of the Maker Buoy PCB.

The Maker Buoy design is open source and both variants described here share the same functional design morphology, hardware components, and software. As such, these common features and components will be described first. The main components are shown in the block diagram in Figure 1A and are briefly described here. The Maker Buoy uses an Adafruit Feather M0 bare microcontroller that is programmed using the Arduino integrated development environment (IDE). GPS position data are acquired using an Adafruit Ultimate GPS and are also used to set the system time upon startup. Other sensors include a nine degree of freedom inertial motion unit (IMU) and an internal temperature sensor. Power is normally provided by a 2000 mAh LiPo battery and is recharged using a solar panel. However, due to variable weather conditions and limited daylight in winter in Greenland, both variants presented here use a 1-Watt solar panel with a 6600 mAh lithium polymer (LiPo) battery to increase the operational lifetime of each buoy. A Watchdog Timer can be used to monitor the microcontroller’s heartbeat signal, and execute a forced reboot if no signal is detected. A light emitting diode (LED) flashes every 30 s to indicate battery voltage and during data collection and transmission. Data are transmitted and received using a Rock7 RockBlock 9603 Iridium modem (http://www.rock7mobile.com/products-rockblock-9603) that uses Iridium’s Short Burst Data (SBD) format. A complete Bill of Materials (BOM) and all design and software files are available at http://github.com/wjpavalko/Maker-Buoy.

Data are stored as little endian hexadecimal and are transmitted in 50 byte packets. The collection time (i.e., the total time that the sensors are powered on and measuring. Default value: 5 min) and the collection interval (i.e., time between measurements. Default value: 30 min) is user-defined and can be changed by sending commands via satellite to the buoy. Satellite communication and access to data in the absence of an internet connection will be described in more detail in Section 2.4 and Appendix A.

**Figure 2:**
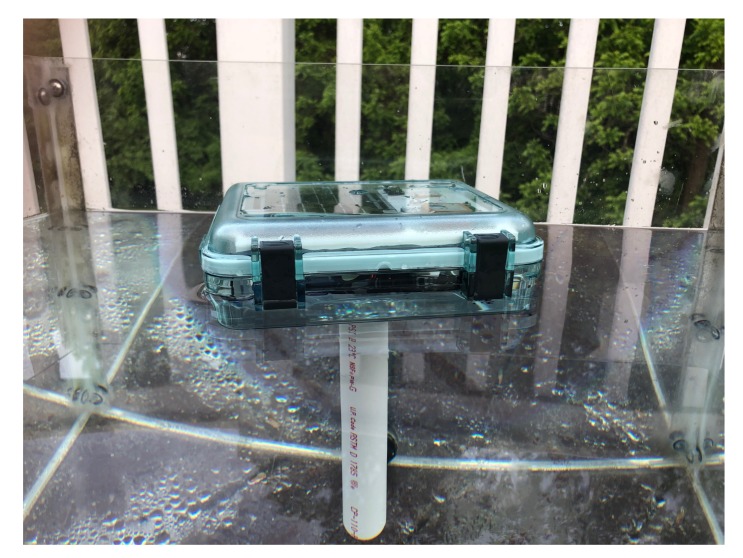
A drifting Maker Buoy without the protective sleeve floating in an aquarium during testing. The PVC stabilizer is visible underwater.

An open source printed circuit board (PCB; Figure 1B,C) is used to connect all electrical components. Electronic components are housed in a waterproof and clear Lexan box with a hinged top that is secured with two clasps. A 3D printed frame fits inside the Lexan box and provides mounting surfaces for all components. The solar panel, GPS, and Iridium modem are mounted on the top panel, just beneath the clear lid. The battery, PCB and soldered components, and LiPo charger are mounted on the bottom plate. The 3D printed legs connecting the top and bottom plates provide enough bottom clearance for a silica desiccant packet to sit on the bottom of the Lexan box, underneath the bottom plate. A stabilizer in the form of a 41.6 cm (16 inch) section of 2.54 cm (1 inch) diameter schedule 40 polyvinyl chloride (PVC) pipe maintains the buoy’s vertical orientation while floating in the water (Figure 2).

### 2.2. GLOF Buoy

**Figure 3:**
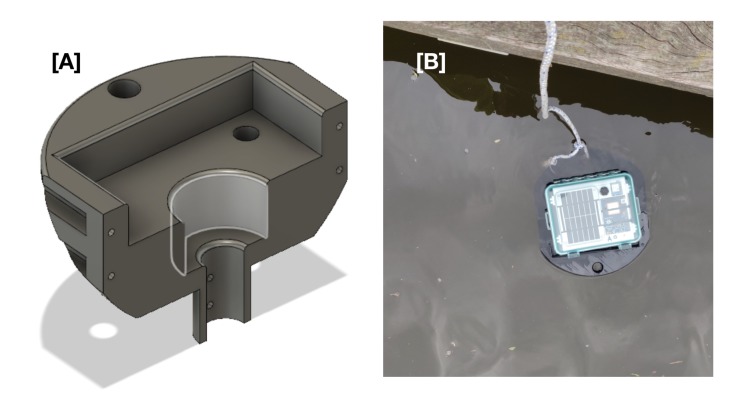
(**A**) One half of the 3D printed sleeve that was designed to protect the Maker Buoy when deployed in an ice-infested glacial lake. (**B**) A GLOF buoy with the outer protective sleeve installed undergoing buoyancy testing in a freshwater stream.

The Maker Buoy was modified to measure water level in an ice-infested glacial lake in southern Greenland. A 3D printed protective sleeve (Figure 3A) was designed to wrap around the Lexan case to protect it from impact and compression, if it was caught between several pieces of ice [31]. The rounded profile should cause the buoy to rise up when squeezed between pieces of ice and the design included large-diameter holes for rope attachments (Figure 3B).

The total size of the sleeve required to completely encase the Lexan box exceeded that of our 3D printer beds. As a result, the protective sleeve was printed in symmetrical halves (Figure 3A) that were secured around the Lexan case and stabilizer pipe using 6 × M6 bolts and marine adhesive. The sleeve design did not interfere with the clasps or hinges on the lid of the Lexan case to enable easy access to the internal components. The sleeve was printed in polyethylene terephthalate glycol (PETG) using 50% infill to provide adequate buoyancy (Figure 3B) while still providing sufficient structural integrity to withstand collisions. A 100 g weight was added to the end of the PVC stabilizer to ensure that the buoy rolled onto its side when on a solid surface. Styrofoam was added to the upper 10 cm of the PVC stabilizer to help offset the additional weight when immersed in freshwater.

The standard Maker Buoy sensors were altered to include a Bosch BMP388 pressure sensor. The BMP388 pressure sensor is low-cost, lightweight, low-power, and has a standard uncertainty of changes in barometric pressure of ± 8 Pa, which would translate to a standard uncertainty of changes in elevation of 0.66 m (by assuming that the air pressure drops approximately by 12 Pa per metre of elevation). The uncertainty in barometric pressure reported by the manufacturer is only valid for temperatures between 25 ${}^{{}^{\circ}}$C to 40 ${}^{{}^{\circ}}$C, requiring testing at lower temperatures. Nevertheless, these properties make it ideal for integration with the Maker Buoy as a water level sensor in a glacial lake, where the lake level is known to increase by 50–100 m during the summer meltwater runoff season. Thus, the water level in the lake can be estimated using vertical movements of a floating Maker Buoy, as recorded by the pressure sensor. In order to extract the vertical movement of the buoy from the pressure measurements, variations due to changes in ambient air pressure were subtracted using observations from a fixed reference station nearby. The reference station was either a weather station or a second Maker Buoy installed on land.

**Figure 4:**
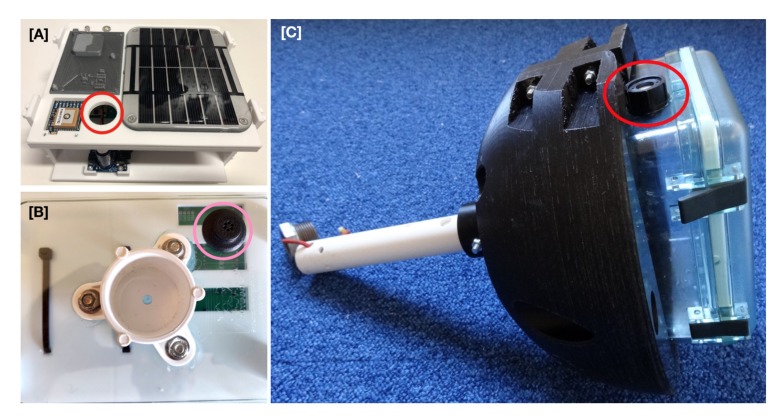
(**A**) The top plate of the 3D printed frame was modified to include a hole for the threaded vent (red circle). (**B**) A snap-in vent was installed on the bottom of the Lexan case (pink circle). (**C**) M16 threaded liquid tight vent (red circle) installed on the side of the Lexan case.

Two liquid tight Heyco vents (Heyco Products, Toms River, NJ) were initially installed in the Lexan case by carefully drilling appropriately-sized holes using either a spade bit or a step bit. A M12 threaded vent was installed on the lid and a snap-in vent was installed on the bottom of the case (Figure 4). Silicone sealant was used as an added precaution. The top plate of the 3D printed Maker Buoy frame was altered to include a hole for the threaded vent. A third liquid tight vent from RS PRO, M16 threaded (RS Components, Copenhagen), was installed on the side of the Lexan case during the last day of field testing (Figure 4), since the response of the pressure sensor to external changes in pressure was slower than expected.

### 2.3. Drifting Buoy

The drifting buoy variant of the Maker Buoy was designed to track objects that were transported by ocean currents, like icebergs, and oceanographic instrumentation. The Maker Buoy was originally designed for a similar purpose, so minimal modifications were required for this use case. Iceberg tracking, however, required additional sensors to determine if the buoy was drifting on its host iceberg, or if it had become detached and was drifting freely, as rolling icebergs can cause trackers to become detached [27]. The transition from iceberg tracker to ocean drifter can be difficult to identify from GPS positions alone [27] and the Maker Buoy’s capability to integrate additional sensors made this change easier to detect.

The standard Maker Buoy was modified to measure average and maximum pitch and external temperature, in addition to GPS position and internal temperature. The drifting buoys were also ballasted to reduce windage. The buoy’s pitch was measured with an accelerometer and provides some indication of sea surface roughness. When drifting freely, ocean surface gravity waves caused the buoy to record large maximum pitch values, while a buoy attached to an iceberg remained relatively stable until the iceberg changed its orientation and/or the buoy fell off the ice. External temperature measurements also aided in identifying a transition from iceberg tracker to surface drifter. While on the iceberg, the buoy recorded air temperature, which exhibited larger diurnal variability than sea surface temperature (SST) in Greenland during summer.

### 2.4. Data Access and Management

The RockBlock 9603 Iridium modems sent data to Rock7 where they could be viewed and/or downloaded in a web browser at https://rockblock.rock7.com/Operations. Here, data distribution was managed by adding ‘Delivery Groups,’ which could be an email address, a web service, or another RockBlock modem. Using an additional RockBlock satellite modem as a delivery group enabled access to position data in remote areas without cellular coverage and the hardware and software required are described in Appendix A.

### 2.5. Hullet Lake

Lake Hullet is one of the largest ice dammed lakes in Southern Greenland and is situated approximately 30 km NE of Narsarsuaq [32]. The lake is fed by meltwater from three glaciers: Nordgletscher, Østgletscher and Sydgletscher. The lake drains mainly through Sydgletscher and further under the Kiattuut Sermiat glacier [33]. Sudden emptying of the lake (GLOF) has been recorded every 1–2 years since 1957 [33]. The lake takes a few weeks to empty, during which time the water level drops approximately 100 m [8]. Given the nearly annual occurrence of GLOFs, Lake Hullet is an ideal test site for the GLOF EWS project. The 30 km distance from the lake to Narsarsuaq prohibited the use of mobile internet and long range wireless devices, like the Fieldserver [34]. Therefore, the GLOF EWS project tested the utility of the Maker Buoy to monitor water level in Lake Hullet.

**Figure 5:**
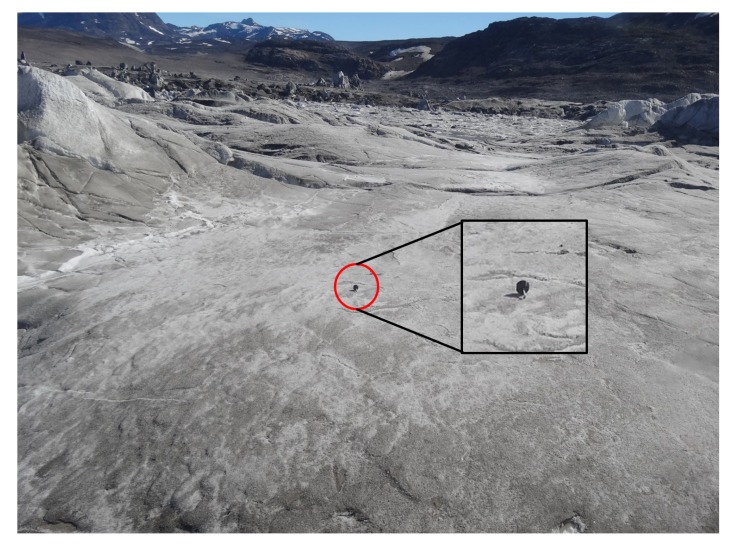
The glacial lake outburst flooding (GLOF) buoy (red circle) deployed on the floating ice in Lake Hullet on 18 June 2019. Inset: an enlargement that shows the buoy resting on the ice after deployment by helicopter.

A field trip to the lake was carried out 17–20 June 2019 to test and deploy two of the new GLOF buoys for water level monitoring (Section 2.2; Figure 5). Further, a previously-installed water level sensor (Baro-Diver, Van Essen Instruments, Delft, The Netherlands) and a timelapse camera were retrieved. In pre-deployment tests, pressure data measured by the buoys were compared to barometric pressure measurements collected with the BaroDiver and a portable precision barometer (DPI 740, GE Sensing / Druck). The two GLOF buoys were configured to record data for 5 min every 60 min, with three sets of observations transmitted via satellite every 180 min. A weather station at Narsarsuaq was used as a reference station for air pressure to remove atmospheric fluctuations.

### 2.6. Vaigat Strait

The Vaigat Strait connects northern Disko Bay and multiple fjord systems, including one of Greenland’s largest outlet glaciers, Jakobshavn Isbræ [35], to Baffin Bay [36]. Many of the icebergs produced at Jakobshavn Isbræ and other marine terminating glaciers in the region enter the Vaigat Strait [35]. Natural oil seeps have been found in the western Vaigat Strait [37] making this region ideal for studies of the impacts of icebergs on microbial oil degradation. Therefore, the western end of the Vaigat Strait was selected for the Vaigat Iceberg—Microbial Oil degradation and Archaeological heritage investigation (VIMOA), which was carried out from 29 July to 16 August, 2019. The multidisciplinary research objectives included investigations of iceberg drift and deterioration, oil degradation by microbes, coastal archaeological sites [38], and testing new technology for iceberg monitoring [39,40].

**Figure 6:**
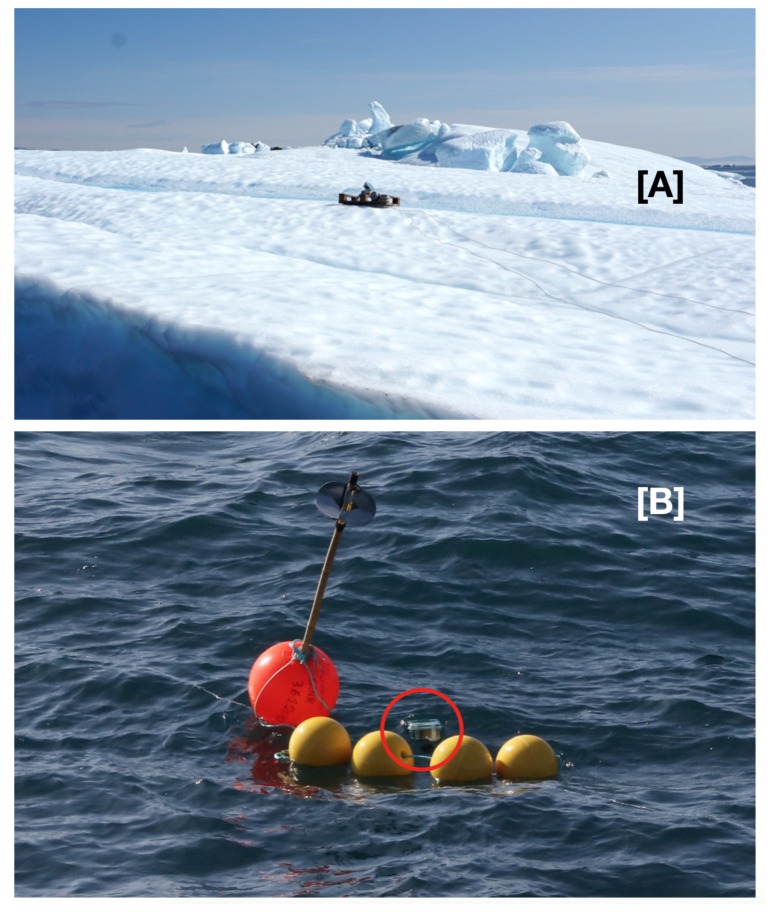
(**A**) A drifting buoy variant of the Maker Buoy was deployed on an iceberg to permit repeat surveys during the Vaigat Iceberg—Microbial Oil degradation and Archaeological heritage investigation (VIMOA) cruise in August 2019 (Photo credit: M.J. Walsh). (**B**) A drifting buoy variant of the Maker Buoy (red circle) was attached to the freely drifting mooring that was deployed and retrieved during the VIMOA cruise in August 2019.

During VIMOA, the deterioration of an iceberg was quantified using low-altitude drone imagery and structure from motion (SfM) photogrammetry software. The deterioration rate, or the rate at which a drifting iceberg loses mass in response to melting and fracturing, can be determined using repeated surveys over the course of several days to a week [29,41]. Estimates of the total iceberg mass can be obtained using the volume of the ice above-water and the density difference between the ice and seawater [29,41]. These mass estimates, however, are only valid when the iceberg is freely drifting. The multidisciplinary nature of the VIMOA cruise required a variety of oceanographic, biological, and chemical sampling to be conducted throughout the western end of the Vaigat Strait and, therefore, the ship could only stay with the target iceberg for a few hours at a time. To enable remote tracking of the target iceberg on board a ship with no internet access, a drifting buoy variant of the Maker Buoy (Section 2.3) was attached to a 200 m long iceberg on 3 August 2019 (Figure 6). The buoy was lashed to a wooden half-pallet that was anchored to the ice using screws (Figure 6). The iceberg buoy was programmed to record data for 5 min every 30 min. Three sets of observations were stored on the microcontroller and these were transmitted every 90 min. The data were retrieved using the methods described in Section 2.4.

Additionally, microbial oil degradation experiments [42] were attached to the iceberg and to a freely drifting mooring, both of which needed to be retrieved before the completion of the research cruise. Thus, a second drifting buoy variant of the Maker Buoy was attached to the drifting buoy (Figure 6B) and the data were also retrieved in near-real-time from the ship.

## 3. Results and Discussion

### 3.1. GLOF Buoys

**Figure 7:**
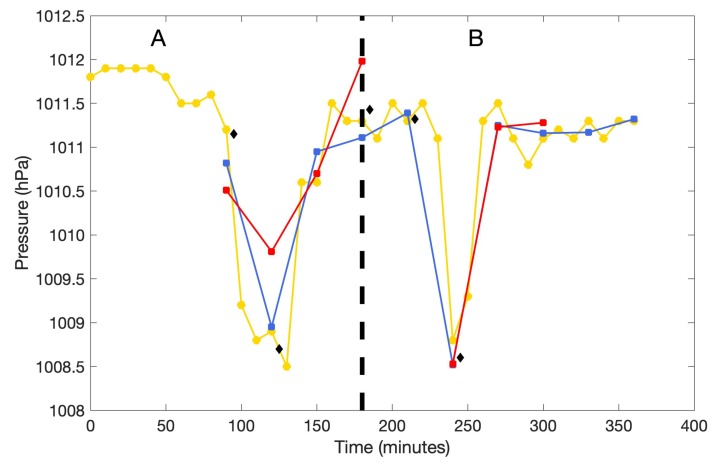
Comparison of pressure data measured by the BaroDiver (yellow), DPI 740 (black diamonds), and the GLOF buoys A and B (red and blue, respectively). The thick dashed line separates the initial test without the extra vent installed (**A**) and testing after extra vent was installed (**B**).

Before deployment in Lake Hullet, the buoys were tested to examine the efficiency of the vents by comparing the barometric pressure measurements with the BaroDiver logger and the DPI 740 portable precision barometer (Section 2.5). One hour after applying power, both buoys were carried uphill until the DPI 740 reported a drop in ambient pressure of 2.5 hPa. At this point, one of the Lexan boxes was opened and the other remained closed. After both buoys collected a measurement the closed box was opened to ensure that it had achieved equilibrium with the ambient pressure. Then, both boxes were closed and were transported back downhill where another measurement was taken, again with one box open and one box closed. Examination of the test data revealed that the buoys reported significantly different pressures when one measured with the box open and the other with the box closed, which suggested that the initial vent installation did not ensure adequate pressure equalization (Figure 7A). Therefore, an extra vent was installed in each box and the test was repeated with much better results (Figure 7B).

**Figure 8:**
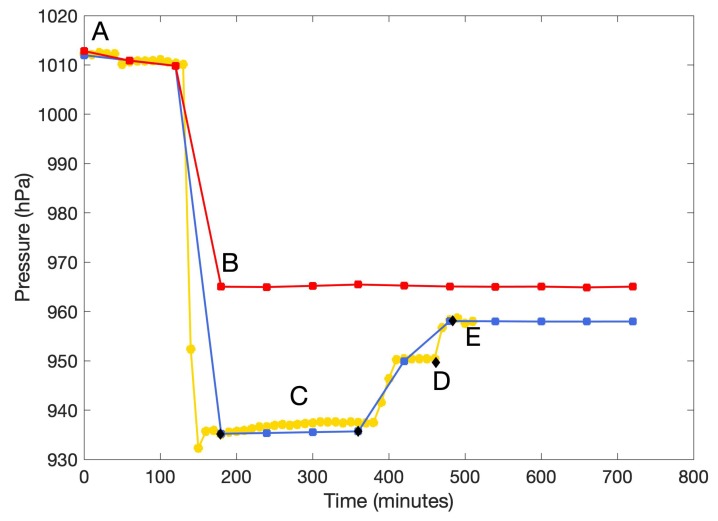
Barometric pressure data from GLOF buoy A (blue line), buoy B (red line), BaroDiver (yellow line) and manual high precision measurements (black dots). (A) Leaving Narsarsuaq close to sea level;( B) deploying buoy B at glacier tongue; (C) leaving buoy A at known reference point for 3 hours, 670 m ASL; (D) buoy A measuring at 546 m ASL; (E) deploying buoy A on dry lake bed, 472 m ASL.

A final test of the performance of the barometric measurements was carried out in the field. GLOF buoy A was initially placed together with the BaroDiver for 3 hours at a reference point at 670 m above sea level (ASL), as previously measured by real-time kinematic (RTK) GPS (Figure 8). Barometric pressure was also manually measured several times using the DPI 740 (Figure 8). From the known reference point, GLOF buoy A and the BaroDiver were moved to a lower elevation (546 m ASL) for a short period, and then to the final deployment site on the dry lake bed at 472 m ASL (Figure 8). The test results revealed that the GLOF buoy pressure measurements were comparable to pressure data from the reference pressure sensors (BaroDiver and DPI 740), and that the GLOF buoy measurements responded well even to sudden pressure changes.

**Figure 9:**
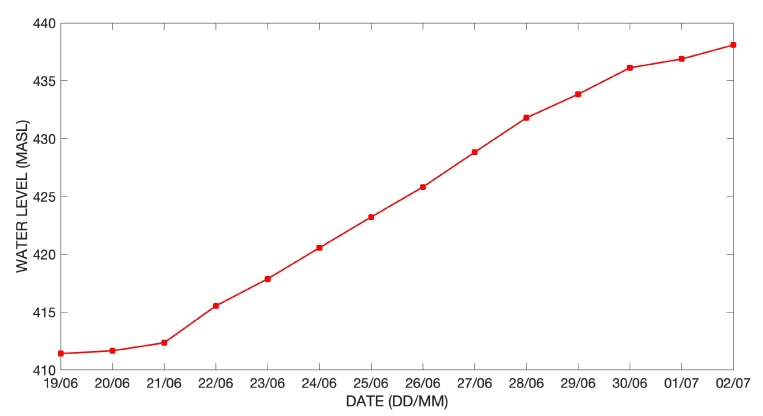
An example of processed water level data (converted from barometric data) from GLOF buoy B used to monitor water level in Lake Hullet. The example data covers the period from 19 June 2019 to 2 July 2019.

After testing, the two GLOF buoys were deployed in the Lake Hullet basin on 18 June 2019. GLOF buoy A was deployed on the dry lake bed as high as possible (472 m ASL) where there were clear signs of silt deposits, indicating flooding within the last year (Figure 8). The risk of ice damage, therefore, was minimized, and Buoy A was used to provide a second set of reference pressure data until it started floating with the rising water level. Once the water level in Lake Hullet reached GLOF buoy A it would start moving, resulting in a significant change in barometric pressure and GPS position, and thereby sending a clear signal that the critical lake level had been reached and that a GLOF event was imminent. GLOF buoy B was deployed from a helicopter onto the floating ice tongue of the terminating glacier (Figure 5 and Figure 8). GLOF buoy B, therefore, continuously tracked water level variability as the inflow of meltwater lifted the ice tongue. The pressure data reported by GLOF buoy B were converted to changes in water level and example data from the first two weeks after deployment show that the water level steadily increased with the inflow of meltwater from the surrounding glaciers (Figure 9). The measured change in water level will be evaluated, along with other data sources, in a future manuscript.

### 3.2. Drifting Buoys

#### 3.2.1. Iceberg tracking

**Figure 10:**
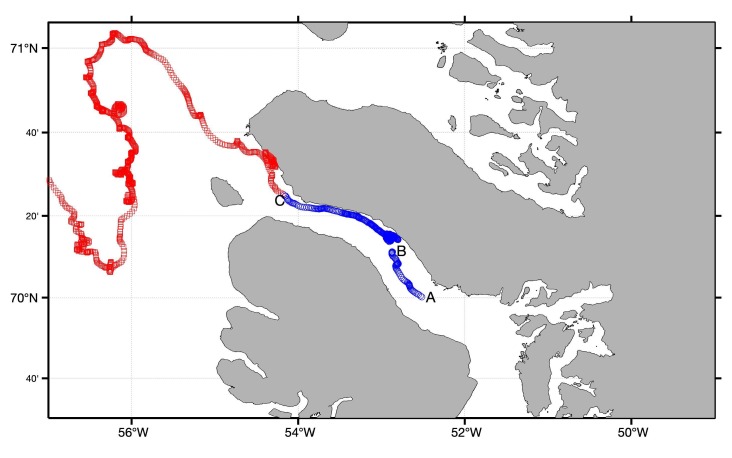
The segments of the buoy’s trajectory on the iceberg and drifting freely are indicated by the blue and red markers, respectively. The buoy was deployed on the iceberg at point A, fell into the water at point B and was reattached to the iceberg, and fell off the iceberg and began drifting unattended at point C.

The iceberg’s trajectory is shown in Figure 10. The buoy was detached from the iceberg on 5 August 2019 (Figure 10), at which point it inverted, preventing GPS data measurements and data transmission. Fortunately, the iceberg and the pallet were located on 6 August 2019. A weight was added to the bottom of the pallet to ensure that it would float upright if pulled from the iceberg. The iceberg drifted until 11 August 2019 when it grounded on a shallow (60 m) sill at the western end of the Vaigat Strait (Figure 10). The buoy fell off the iceberg on 12 August 2019 and drifted westwards into Baffin Bay.

**Figure 11:**
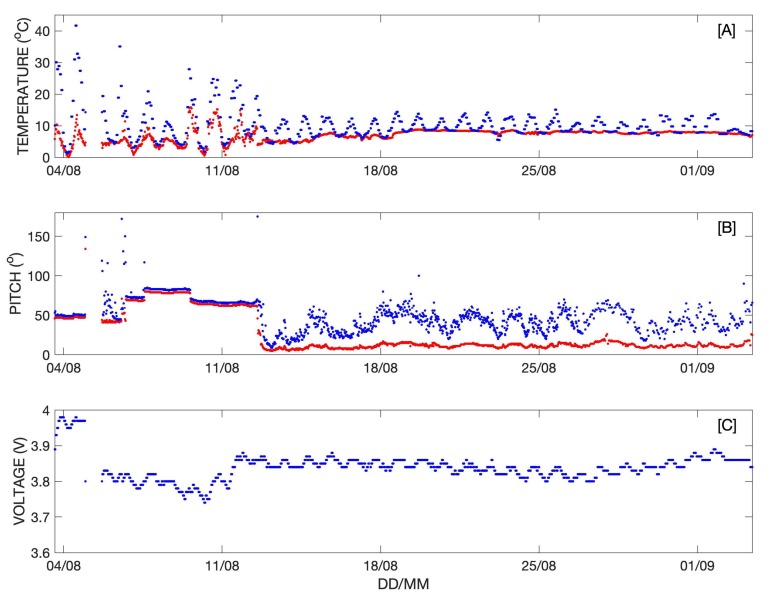
(**A**) External and internal temperature are indicated by red and blue dots, respectively. (**B**) Average and maximum pitch are indicated by red and blue dots, respectively. (**C**) Battery voltage.

Time series of the temperature (internal and external), pitch, and battery voltage are shown in Figure 11. The internal and external temperature and pitch measurements enabled accurate identification of the transition from iceberg tracker to surface drifter (Figure 11A). While on the iceberg, the external temperature sensor recorded ambient air temperature, which varied from approximately 0 ${}^{{}^{\circ}}$C to 15 ${}^{{}^{\circ}}$C (Figure 11A). The internal temperature often exceeded 20 ${}^{{}^{\circ}}$C at mid-day (Figure 11A). This changed when the buoy fell into the water on 12 August as the prominent diurnal cycle seen in air temperature was no longer evident in the external temperature data (Figure 11A). The diurnal cycle persisted in the internal temperature data, but the daily maxima decreased significantly due to the cooling effect of the seawater (Figure 11A).

Similarly, while on the iceberg the average and maximum pitch measurements were similar in magnitude, though step-wise changes in pitch were observed that were probably due to changes in iceberg orientation following small calving events that changed the iceberg’s center of mass (Figure 11B). After transitioning to a surface drifter, the average pitch data indicated a nearly vertical orientation with considerable variability observed in the maximum pitch, likely caused by surface gravity waves (Figure 11B). The battery voltage time series shows that the time spent inverted in the water drained the battery as the buoy attempted to collect and transmit data with its solar panels obscured (Figure 11C). A period of persistent fog and the nearly horizontal orientation of the buoy while on the iceberg limited the ability of the solar panel to fully recharge the battery.

Combining temperature and pitch data with the GPS positions enabled accurate identification of the transition from iceberg tracker to surface drifter, a task that proved difficult in a previous study of iceberg drift that used the Expendable Ice Tracker (EXITE) [27]. The EXITE uses the Spot Trace GPS asset tracker, which is relatively low cost, but it cannot transmit data from additional sensors (like temperature and pitch) and an internet connection is required to view the data. Additionally, the Spot Trace uses the Global Star constellation of satellites, which lacks the global coverage offered by Iridium.

Near-real-time access to the iceberg’s position allowed the research vessel to locate it, even in dense fog. As a result, the iceberg was revisited three times during an eight-day period. Aerial drone SfM surveys were performed each time the iceberg was located and the oil degradation mooring was successfully retrieved. The results of the drone SfM surveys and oil degradation moorings will be reported in future manuscripts.

#### 3.2.2. Drifting Mooring Tracking

**Figure 12:**
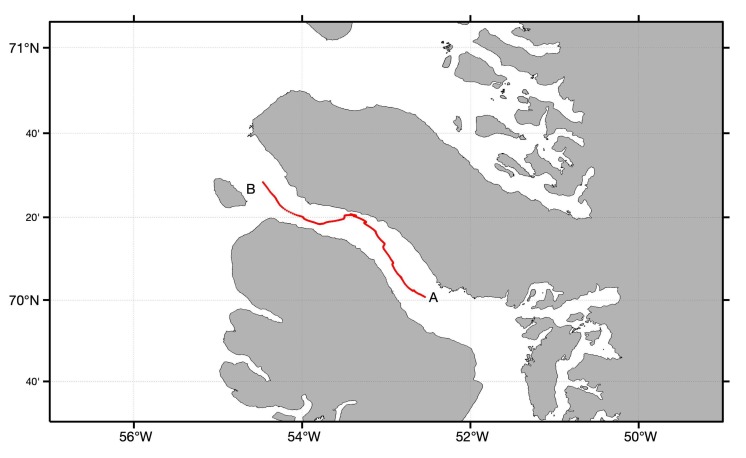
The drifting mooring’s trajectory through the Vaigat Strait from 4–12 August 2019. The mooring was deployed at point A and retrieved at point B.

The freely drifting mooring was deployed 4 August 2019 during the VIMOA cruise. The drifting mooring consisted of surface flotation, oil degradation experiments distributed over 100 m of rope, and a weight. A drifting buoy variant of the Maker Buoy, identical to the buoy that was used to track the iceberg (Section 3.2.1), was attached to the surface float (Figure 6) and the data that it transmitted were included in the ‘Field Data’ delivery group (Section 2.4). The drifting mooring’s trajectory is shown in Figure 12). The drifting mooring was successfully retrieved 11 August 2019 at the western end of the Vaigat Strait (Point B in Figure 12).

**Figure 13:**
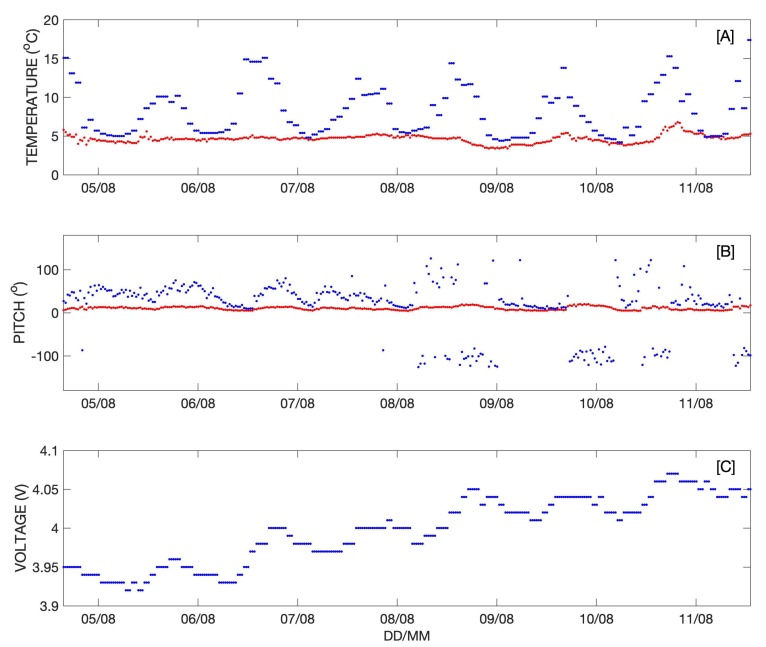
Time series of data recorded by the drifting buoy in the Vagait Strait. (**A**) External (sea surface temperature) and internal temperature are indicated by red and blue markers, respectively. (**B**) Average and maximum pitch are indicated by red and blue markers, respectively. (**C**) Battery voltage.

In addition to recording location information, the temperature and pitch data (Figure 13) provide valuable information about surface ocean conditions. Sea surface temperature (SST) was relatively constant at approximately 5 ${}^{{}^{\circ}}$C (Figure 13) until 8 August, when it became more variable. A similar transition in the maximum pitch data occurred at roughly the same time. The pitch data are indicative of periods of energetic surface gravity waves that were driven by strong winds and the corresponding variability in SST was likely due to wind and wave mixing. The time series of battery voltage shows an increasing trend overall (Figure 13).

## 4. Summary

Two variants of the Maker Buoy were developed to perform unattended environmental monitoring in harsh, remote areas of Greenland. The data were accessible to researchers in near-real-time, both via a standard internet connection and in the field. The field campaigns conducted during the summer of 2019 demonstrate the potential for low-cost and open source platforms like the Maker Buoy to monitor water levels in glacial lakes and to track drifting icebergs, oceanographic instrumentation, and surface ocean currents. Given the successful deployment in Lake Hullet, the Maker Buoy may also be useful in monitoring water levels in glacial lakes elsewhere in Greenland, as well as in other countries, and may provide ground truth data to validate remote sensing measurements [43,44]. The two variants of the Maker Buoy presented here, therefore, may provide a valuable low-cost monitoring tool for both scientists and resource managers.

## Figures and Tables

**Figure A1:**
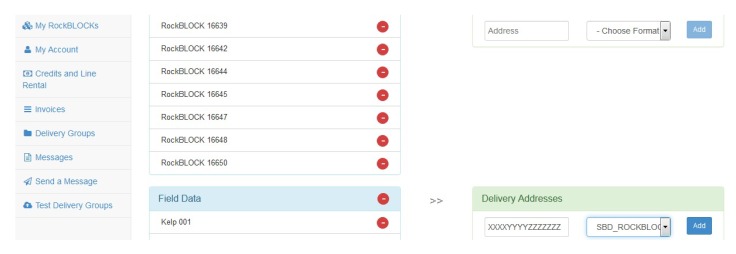
A screenshot of the ‘Delivery Group’ page on the Rock7 website (https://rockblock.rock7.com/Operations).

**Figure A2:**
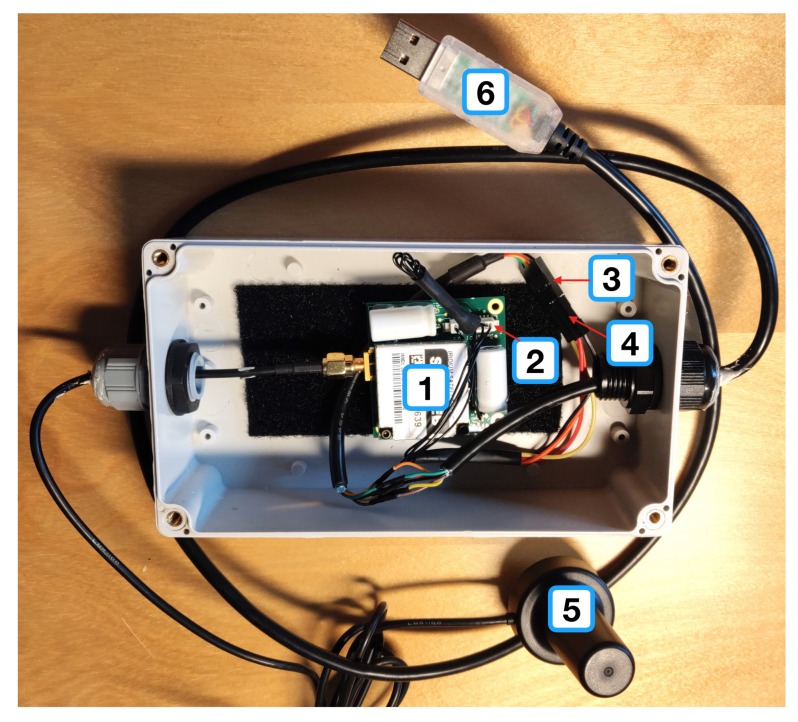
A RockBlock 9603 Iridium modem was housed in a PVC junction box (shown here with the lid removed) and was used to retrieve data in the field. The numbering corresponds to: 1. RockBlock 9603 Iridium modem; 2. Picoblade cable; 3. Female jumper cable; 4. Male jumper cables; 5; Maxtenna external SMA Iridium antenna; 6. FTDI serial TTL-232 USB cable.

## Abbreviations

The following abbreviations are used in this manuscript:

°	Degrees
3D	Three-dimensional
ASCII	American Standard Code for Information Interchange
ASL	Above Sea Level
BOM	Bill of Materials
°C	Degrees Celsius
EWS	Early Warning System
EXITE	Expendable Ice Tracker
FTDI	Future Technology Devices International
GLOF	Glacial Lake Outburst Flood
GPS	Global Positioning System
GrIS	Greenland Ice Sheet
hPa	Hecto-Pascal
IDE	Integrated Development Environment
IMEI	International Mobile Equipment Identity
IMU	Inertial Motion Unit
km	Kilometer
LED	Light Emitting Diode
LiPo	Lithium Polymer
m	Meter
mAh	Milli-amp Hour
min	Minute
PCB	Printed Circuit Board
PETG	Polyethylene Terephthalate Glycol
PVC	Polyvinyl Chloride
RTK	Real-Time Kinematic
SBD	Short-Burst Data
SfM	Structure from Motion
SMA	SubMiniature version A
SST	Sea Surface Temperature
SWAP	Size, Weight, and Power
TTL	Transistor-Transistor Logic
USB	Universal Serial Bus
V	Volts
VIMOA	Vaigat Iceberg Microbial Oil degradation and Archaeological heritage investigation

## Appendix A. Field Data Access

The delivery group(s) must be configured on the Rock7 website before departing for the field. Log in to your account and navigate to ‘Delivery Groups.’ Under ‘Add Delivery Group,’ create a name for the data that require monitoring in the field. Here, we call this delivery group ‘Field Data’ (Figure A1) and we have added one drifting buoy ‘Kelp 001.’ Next, add the international mobile equipment identity (IMEI) number of the RockBlock modem that will be used to retrieve the data and select the ‘SBD_ROCKBLOCK’ format in the drop-down menu (Figure A1). Finally, click on the ’add’ button to create the delivery group and messages from the selected buoys will be sent to your field RockBlock and stored in a queue.

To access the data that are stored in the queue for your field RockBlock an additional RockBlock 9603 Iridium modem must be connected to a computer. This requires a Picoblade 10 circuit 150 mm cable, M/F jumper wires (5.91”), an external Iridium antenna with a subminiature version A (SMA) connector, and a Future Technology Devices International (FTDI) serial transistor-transistor logic (TTL)-232 universal serial bus (USB) cable with bare leads (Figure A2). The Picoblade cables were soldered to the male jumper cables, which were inserted into the female receiver, which was then soldered to the FTDI TTL-232 USB cable and all of the components were housed in a PVC junction box for weather-proofing (Figure A2). The external Iridium antenna cable was routed through a M16 cable gland and was screwed on to the SMA attachment on the RockBlock 9603. The USB cable was routed through a M12 cable gland and connected to a laptop computer. Using the external Iridium modem increased the length of the overall assembly, which allowed the laptop computer and operator to remain indoors and out of the elements. Users with a rugged laptop could opt for the RockBlock 9603 modem with the patch antenna, as used on the buoys.

Any computer that supports FTDI serial to USB drivers can be used to communicate with the RockBlock modem though here we used a Windows laptop. After installing the FTDI drivers and configuring the serial port, plug the USB cable into the appropriate port on your computer. In this example, commands are sent and retrieved using RealTerm (https://realterm.sourceforge.io/), a free serial terminal software. Instructions for retrieving data via a serial connection are described in the RockBlock documentation (https://docs.rockblock.rock7.com/docs/receive-data). Here, we describe the steps required to retrieve data using RealTerm:

- Click on the ‘port’ tab
- On the ‘Port’ drop-down menu - select \VCP0
- Select 19200 baud rate
- Click ‘open’ twice
- Click on the ‘send’ tab
- In the top left dialogue box enter the following commands, followed by ‘Send ASCII’
- Check modem communication: AT\r
  – Response: OK\r
- Turn off Flow Control: AT\&K0
  – Response: OK\r
- Start short burst data (SBD) session: AT+SBDIX\r
  – Response format: +SBDIX: transmit status, message number (TX), message status, message number (RX), message length, number of queued messages
  – transmit status: A value between 0-2 indicates that your AT+SBDIX\r message was received while any number >2 indicates that your message was not received.
  – message number (TX): The number of the message (AT+SBDIX\r), which can be ignored
  – message status: A number between 0-2
    ∗ 0: No messages waiting
    ∗ 1: New message retrieved
    ∗ 2: Error
  – message number (RX): Number of the message received, which can be ignored
  – message length: Length of the message, in bytes
  – number of queued messages: self-explanatory
- If your message was not received (transmit status >2), make sure that the antenna has an unimpeded view to the sky and send AT+SBDIX\r again
- If transmit status is between 0-2, navigate to the ‘Capture’ tab in RealTerm
- Enter the desired directory and file name in the file dialogue box
- Check the box that reads ‘Capture as Hex’
- Click either ‘Start: Overwrite’ or ‘Start: Append’
- Navigate to the ‘Send’ tab in RealTerm
- Enter AT+SBDRB\r and click ‘Send ASCII’
- Navigate to the ‘Capture’ tab in RealTerm and click ‘Stop Capture’
- Open the file that was created from your RealTerm session and verify the contents. Note that the first 11 HEX characters in the capture file correspond to AT+SBDRB<CR> followed by a two-byte HEX containing the message length
- Download any additional messages by repeating this process
- Once finished, navigate to the ‘Port’ tab in RealTerm and click on ‘Open’ to close the port

Note that all messages transmitted from buoys will be sent to the queue for the field RockBlock, with the oldest message first. All messages in the queue must be downloaded to access the most recent data. Alternatively the queue can be flushed by sending the following commands in RealTerm:

- Open RealTerm and connect to the field RockBlock by following the instructions above
- Send the following commands, followed by ‘Send ASCII’
  – AT\r
  – AT\&K0
  – AT+SBDIX\r
  – AT+SBDWT=FLUSH_MT\r
  – AT+SBDIX\r

After flushing the message queue, wait until the next scheduled transmission time to download the most recent data.
